# Probiotics Interact With Lipids Metabolism and Affect Gut Health

**DOI:** 10.3389/fnut.2022.917043

**Published:** 2022-05-31

**Authors:** Taoying Wu, Guangqiang Wang, Zhiqiang Xiong, Yongjun Xia, Xin Song, Hui Zhang, Yan Wu, Lianzhong Ai

**Affiliations:** ^1^Shanghai Engineering Research Center of Food Microbiology, School of Health Science and Engineering, University of Shanghai for Science and Technology, Shanghai, China; ^2^Hunan Key Laboratory of Bean Products Processing and Safety Control, School of Food and Chemical Engineering, Shaoyang University, Shaoyang, China; ^3^Department of Food Science and Technology, School of Agriculture and Biology, Shanghai Jiao Tong University, Shanghai, China

**Keywords:** inflammatory bowel disease, bowel cancer, lipid metabolism, gut microbiota, probiotics

## Abstract

Probiotics have attracted much attention due to their ability to modulate host intestinal microbe, participate in nutrient metabolism or immunomodulatory. Both inflammatory bowel disease (IBD) and bowel cancer are digestive system disease, which have become a global public health problem due to their unclear etiology, difficult to cure, and repeated attacks. Disturbed gut microbiota and abnormal lipid metabolism would increase the risk of intestinal inflammation. However, the link between lipid metabolism, probiotics, and IBD is unclear. In this review, we found that different lipids and their derivatives have different effects on IBD and gut microbes. ω-3 polyunsaturated fatty acids (PUFAs) docosahexaenoic acid, eicosapentaenoic acid, and their derivatives resolvin E1, resolvin D can inhibit oxidative stress and reactive oxygen species activate NFκB and MAPk pathway. While ω-6 PUFAs linoleic acid and arachidonic acid can be derived into leukotrienes and prostaglandins, which will aggravate IBD. Cholesterol can be converted into bile acids to promote lipid absorption and affect microbial survival and colonization. At the same time, it is affected by microbial bile salt hydrolase to regulate blood lipids. Low denstiy lipoprotein (LDL) is easily converted into oxidized LDL, thereby promoting inflammation, while high denstiy lipoprotein (HDL) has the opposite effect. Probiotics compete with intestinal microorganisms for nutrients or ecological sites and thus affect the structure of intestinal microbiota. Moreover, microbial short chain fatty acids, bile salt hydrolase, superoxide dismutase, glutathione, etc. can affect lipid metabolism and IBD. In conclusion, probiotics are directly or indirectly involved in lipids metabolism and their impact on IBD, which provides the possibility to explore the role of probiotics in improving gut health.

## Introduction

The intestinal tract is an immune organ for human protection, and it is also an important place for the digestion and absorption of dietary nutrients. Bacterial infection, viral infection, immune imbalance, etc. would induce inflammatory bowel disease (IBD) (1–3). IBD generally does not lead to bowel cancer, but it will increase the incidence of bowel cancer in patients with ulcerative colitis (UC), but without Crohn’s disease (CD) (3, 4). In recent years, the incidence of bowel cancer has increased year by year and shows a younger trend, which is a serious worldwide health problem (5). More and more studies have shown that although the occurrence of bowel cancer is related to genetic factors, it is mainly caused by polluted environment, pathogenic bacteria and toxins, bad dietary and lifestyle (6–9). Intestinal microbe and nutrient metabolism play an important role in controlling host immunity and maintaining intestinal homeostasis (10). However, intestinal dysbiosis and metabolic disorders would promote the occurrence and development of bowel cancer (11, 12). Therefore, gut microbiota and dietary nutrition have always been hotspots in cancer research. For a long time, dietary lipid has been considered as a main dietary risk factor for cancer, and it is closely related to IBD and bowel cancer (12, 13).

Dietary lipid is one of three major nutrients in the human body. It is decomposed into fatty acids, cholesterol and other fat digestion products by the action of various enzymes and bile salts in the small intestine, and then absorbed into the bloodstream through the small intestine wall. In previous studies, it was generally believed that high-fat diet was related to the occurrence of certain tumors, and now it is clearer that this is related to the types of lipids and their metabolites (14). In some animal experiments, it was found that ω-3 polyunsaturated fatty acids (PUFAs) can inhibit the occurrence and development of certain tumors, but the effect of ω-6 PUFAs is the opposite (15, 16). At the same time, body lipid is converted into substances needed by the body under the action of various enzymes to ensure the operation of normal physiological functions. However, lipid metabolites may also affect the body’s health, especially in metabolic disorders or immunodeficiency. For example, the PUFAs metabolites such as leukotrienes (LTs), prostaglandins (PGs), and peroxidized linoleic acid (13-HPODE) would promote the production of inflammatory factors, thereby increasing the risk of IBD and bowel cancer (16, 17). Lipid metabolism is a complex and important biochemical reaction process in the body. It is unknown whether it is affected by gut microbes or leads to changes in the species, quantity, proportion, localization, and biological characteristics of intestinal flora, thereby affecting human health.

With the in-depth study of human gut microbes, it has become a consensus that gut microbes can regulate human physiology and metabolism. Moreover, probiotics have attracted extensive attention due to their beneficial effects on the human body. Probiotics are live microorganisms which, when administered in adequate amounts, confer a health benefit to the host. The published results show that probiotics perform well in promoting nutrient absorption, maintaining intestinal homeostasis, and enhancing immunity (18–22). For example, *Lacticaseibacillus casei* LC2W can inhibit the colonization of *Escherichia coli* O157:H7 *in vivo* and reduce the severity of colitis, and *Lactiplantibacillus plantarum* AR326 with good adhesion ability can be used as promising probiotics to ameliorate dextran sulfate sodium (DSS) -induced colitis (23–25). That is to say, probiotics play important roles in down-regulating the production of pro-inflammatory cytokines, promoting intestinal epithelial barrier function, increasing the anti-inflammatory response, and contributing to the overall health of the host (21–25). As important health-promoting bacteria, whether probiotics are involved in lipid metabolism and its mechanism is unclear. In this review, we analyze the effects of lipids and their metabolites on IBD and bowel cancer, and summarize the pathways of probiotics regulating microbiota and participating lipid metabolism, in order to exploring the potential of probiotics in regulating host immunity and maintaining intestinal homeostasis.

## Inflammatory Bowel Disease and Bowel Cancer Seriously Damage Health

IBD is a chronic disease of the digestive system with unclear etiology, difficult to cure, and repeated attacks. It is accompanied by a variety of complications, which seriously affects the quality of life of patients (26). The incidence of IBD has increased significantly in recent years, and there is a younger trend. To date, the exact causes of IBD have not been fully understood and effective therapeutics are yet to be discovered. With regards to etiology, there are pediatric data, mainly from case-control studies, which suggest that some dietary habits, such as consumption of animal protein, fatty foods, high sugar intake, may contribute to IBD onset (27). Poor dietary habits can lead to changes in intestinal flora, which are inseparable from the occurrence of IBD. Fortunately, oral probiotics *Bifidobacterium and Lactobacillus* and fecal microbiota transplantation can restore the diversity of patients’ intestinal flora, which has important clinical value for the treatment of IBD (28–30).

As we known, nuclear factor kappa-B (NF-κB) is a hub for pro-inflammatory transcription factors that induce the expression of many pro-inflammatory genes, such as cell adhesion molecules, cyclooxygenase 2 (COX-2), inducible nitric oxide synthase (iNOS), tumor necrosis factor α (TNF-α), nuclear factor-kappa B (NF-kappaB), and interleukin (IL) (31–33). When endotoxin interacts with Toll-like receptor 4 (TLR4), TNF-α interacts with tumor necrosis factor receptor (TNFR), it will activate the κB kinase (IKK) complex of MAPK/EKK kinase, promote the dissociation of phosphorylated NK-beta inhibitors (IKBα) from the IKBα/NF-κB complex and produce NF-κB monomers, which translocate into the nucleus and produce pro-inflammatory factors, such as TNF-α, IL-1β, IL-6, and IFN-γ) and inflammatory markers (iNOS and COX-2) (34, 35). Similarly, lipopolysaccharide (LPS) and TNF-α bind to receptors TLR4 and TNFR, respectively, which can promote the phosphorylation of the transforming growth factor 1 (TAK1), activate the IKBα/NF-κB and MKK4/JUK pathways, and produce inflammation (36). Studies have shown that IBD more than doubles an individual’s lifetime risk of developing bowel cancer, and the risk increases significantly if they have suffered with UC for a sustained period of time (37). In other words, general intestinal inflammation will not cause bowel cancer, but with the increase of UC time, there will be a certain probability rate of developing colon cancer.

Bowel cancer is the most common digestive tract tumor, including colorectal cancer and small bowel cancer. Colorectal cancer is divided into colon cancer and rectal cancer, while small bowel cancer can be divided into duodenal cancer, ileal cancer and jejunum cancer. Moreover, colorectal cancer is more common than small bowel cancer (38). Colorectal cancer is the third most common cancer worldwide, especially in developed countries where an estimated 60% of all cases occur (39). Generally speaking, genetics, inflammation, intestinal adenomas, dietary habits, and carcinogens are all contributing factors to bowel cancer. Gut microbiota participates in many physiological and pathological processes of the host, including food digestion and absorption, substance metabolism, host immunity, and intestinal inflammation (40–43). It is established that physical inactivity, obesity and some dietary factors (red/processed meats, alcohol) are positively associated with colorectal cancer, while healthy lifestyle habits show inverse associations (6, 9, 44). The type and quantity of food ingested are controllable, thereby affecting the supply of nutrients and the composition of microorganisms. Therefore, dietary factor is one of the most important and controllable factors in changing gut microbes (9). Therefore, more and more scientists are beginning to pay attention to the impact of lipids and their metabolites, and gut microbiota especially probiotics on IBD and bowel cancer.

## Lipid Metabolism Differentially Affect Inflammatory Bowel Disease and Bowel Cancer

### Polyunsaturated Fatty Acids and Cholesterol Relieve Inflammation or Not

**FIGURE 1:**
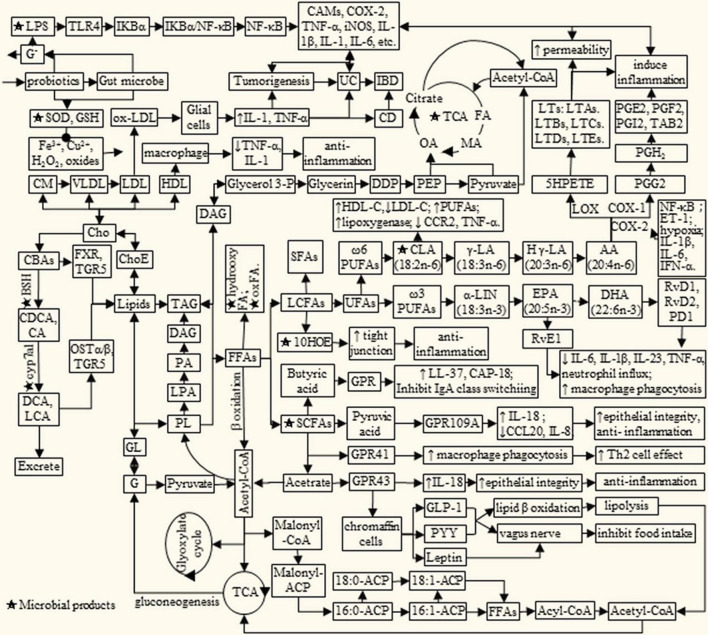
Lipids metabolism interacts with microbial products to influence IBD/cancer. AA, arachidonic acid; BAs, bile acids; BSH, bile salt hydrolase; CA, cholic acid; cAMP, adenosine cyclophosphate; CAMs, cell adhesion molecule; CBAs, conjugated bile acid; CCL20, Chemokine Ligand 20; CD, Crohn’s disease; CDCA, chenodeoxycholic acid; Cho, cholesterol; ChoE, cholesterol ester; CLA, conjugated linoleic acid; CM, chylomicrons; cyp7a1, cholesterol 7α-dehydroxylase; C${}_{C\,a}^{2+}$, concentration of Ca^2 +^; DDP, dihydroxyacetone phosphate; DCA, deoxycholic acid; DHA, docosahexaenoic acid; EPA, eicosapentaenoic acid; ET-1, endothelin-1; FA, fumaric acid; FFAs, free fat acids; FXR, farnesoid X receptor; G, glucose; Glycerol 3-P, glycerol 3-phosphate; GL, glycosphingolipid; GLP-1, lucagon-like peptide-1; GLP-2, glucagon-like peptide-2; GPR, G protein-coupled receptors; HLD, high density lipoprotein; H γ-LA, homologous γ linolenic acid; IB, D, inflammatory bowel disease; IL, interleukin; LA, linoleic acid; LCA, lithocholic acid; LDL, low density lipoprotein; Le, lenoleic; LNA, linolenic acid; LPS, lipopoly saccharide; LTs, leukotrienes; MA, malic acid; Malonyl-CoA, malonyl coenzyme A; ACP, acyl carrier protein; OA, oxalacetic acid; OSTα/β, organic solutetransport protein α/β heterodimer; PA, pyruvic acid; PD1, protectin D1; PEP, phosphoenolpyruvate; PG, prostaglandin; PL, phospholipids; PYY, peptide tyrosine; RvE1, resolvin E1; RvD, resolvin D; SCFA, short chain fatty acid; TAG, triglyceride; TCA, tricarboxylic acid cycle; TLR4, Toll-likereceptor 4; TGR5, G protein coupled bile acidreceptor; UC, ulcerative colitis; VLDL, very low density lipoprotein; COX2, cyclooxygenase 2; 10HOE, 10-hydroxy-cis-12-otadecenoic acid.

Dietary lipids are mainly composed of triglycerides, cholesterol, phospholipids, and glycolipids, which can be digested and absorbed by the body. Lipid metabolism is a complex process, which is affected by many factors. Meanwhile, lipid metabolites will in turn affect the health of the body (Figure 1). Among them, PUFAs and cholesterol have attracted widespread attention due to their association with cardiovascular and cerebrovascular diseases, metabolic diseases, and inflammation. For example, ω-3 PUFAs α linolenic acid can be converted into docosahexaenoic acid (DHA), eicosapentaenoic acid (EPA) and prostaglandin (45). While biologically active derivatives of the ω-6 PUFAs arachidonic acid (AA) include PGs, thromboxane, hydroxyeicosatetraenoic acids and LTs (16, 17, 46, 47). It is reported that dietary supplementation with EPA and DHA decreased low-density lipoprotein cholesterol (LDL-C) synthesis and increased bile acid synthesis and LDL-C clearance by LDL receptor, synergistically with estrogen (48). LTs are potent pre-inflammatory mediators involved in the pathogenesis of inflammation, allergy, asthma, and shock (47).

PUFAs is a component of the plasma membrane, which affects the fluidity of the cell membrane and the transmission of cell signals, regulates a variety of enzymes related to lipid metabolism, and participates in the regulation of inflammation and immunity in the body (49–51). For example, activation of phosphatase A2 enables the release of AA from membrane phospholipids, enhances the phosphorylation of NK-IKBα by κB kinase, and promotes inflammation (17, 47). COX-2 plays a key role in the regulation of inflammation by catalyzing the oxygenation of AA to PGs and hydroperoxides (51, 52). However, ω-3 PUFAs exert anti-inflammatory and antioxidant effects by inducing the production of bioactive compounds, such as 17, 18-epoxyeicosatetraenoic acid, resolvin E1 (RvE1), protectin D1, adiponectin, lysis, interleukin 10 (IL-10), interleukin 4 (IL-4), superoxide dismutase (SOD), heme oxygenase-1 (HO-1), and glutathione (GSH) (51–53). DHA can activate TAK1-binding protein 1 through GPR120/β-arrestin2, preventing the activation of IKBα/NF-κB and MKK4/JUK, thereby inhibiting inflammation (49, 50). Moreover, RvE1 is a derivative of EPA, which blocks transient receptor potential vanilloid type -1 and TNF-α signaling and relieves inflammation. Taken together, ω-3 PUFAs can down-regulate endotoxin- and cytokine-induced expression of COX-2 and lipid peroxidase, and inhibit phospholipase activity, thereby reducing AA and its induced eicosanoids content of inflammatory mediators (54).

Blood lipids are the general term for neutral fats and esters in plasma, which are widely present in the human body. Generally speaking, the main components of blood lipids are triglycerides and cholesterol, among which triglycerides are involved in energy metabolism in the human body, while cholesterol is mainly used for the synthesis of cell plasma membrane, steroid hormones and bile acids. We often use total triglycerides, total cholesterol, LDL and high-density lipoprotein (HDL) as the investigation indicators of blood lipids (55). LDL is a lipoprotein particle that carries cholesterol into peripheral tissue cells and can be converted to the oxidized LDL (ox-LDL), which plays a central role in atherosclerosis by acting on multiple cells such as endothelial cells, macrophages, platelets, fibroblasts, and smooth muscle cells through ox-LDL receptor (LOX-1) (56). While ω-3 PUFAs inhibited hepatic endoplasmic reticulum stress induced by feeding of a high-saturated fat diet accompanied by the expression hem agglutinin-like ox-LDL receptor (LOX-1) (57). In contrast, HDL mediates reverse cholesterol transport and is known to be protective against atherosclerosis (58). In addition, HDL mediates anti-inflammatory reprogramming of macrophages *via* the transcriptional regulator activating transcription factor 3 (ATF3) (59).

### Lipid Oxides Exacerbate Inflammation and Tumors

Oxidation is a normal phenomenon in nature. When it occurs in the human body, it is called biological oxidation, which is realized under the catalysis of enzymes. Lipid oxidation generally includes auto- and enzymatic oxidation, which provides energy and produces various metabolites such as lipid peroxides, alcohols, aldehydes, and ketones. It has be reported that the occurrence and development of multiple chronic diseases such as atherosclerosis, inflammation and cellular aging, are closely related to lipid oxides (60–62). As we known, lipid peroxides are only the unstable reaction intermediates, which react with almost all molecules or cells in the human body, destroying DNA, and cellular structures. PUFA is one of the indispensable and easily oxidized fatty acids in our body. Dietary intakes rich in unsaturated fatty acids, have increased dramatically in recent decades. It is suggested that PUFA intake is beneficial to human health, but some lipid peroxides would increase the risk of inflammation and related diseases (53, 57). The final products of lipid peroxidation are reactive aldehydes such as malondialdehyde (MDA) and 4-hydroxynonenal (HNE), which play important pathogenic roles in the aging-related disorders characterized by increases of oxidative stress (63). Recently, Zhang and colleagues demonstrated that the linoleic acid-rich diet had little effect on the severity of colitis in the treated IL-10^–/–^ mice (64). They also found that oxidized linoleic acid had little effect on basal inflammation in mice, but exacerbated chemically induced colitis in mice (65). Moreover, they isolated and characterized epoxyketooctadecenoic acid (EKODE), which was a lipid peroxidation product, and was among the most dramatically increased lipid molecules in the colon of azoxymethane/dextran sodium sulfate-induced colorectal cancer mice, and exacerbated colonic inflammation and colon tumor genesis (66). In addition, most of the excessive LDL in the blood are oxidized and modified lipoproteins, which are retained in the blood vessel wall and involved in the formation of atherosclerosis (58, 60). Therefore, we should try to prevent and reduce the oxidized lipid to avoid the occurrence and development of IBD and bowel cancer.

### Lipid Metabolites Interact With Gut Microbiota to Relieve Inflammation

There are many kinds of intestinal microorganisms, which are related to age, body weight, gender, region, and dietary. The structure of the intestinal microbiota is seriously influenced by dietary habits. Therefore, the core dominant microbiota in the human gut is related to the proportions of protein, fat and carbohydrate components in dietary patterns (44, 67). At the same time, microorganisms will in turn act on the lipid metabolism of the host. It is reported that probiotics affect the lipid profile and anthropometric indices in diabetic nephropathy (68). And alterations in the gut microbiota of mice induced by a high-fat diet led to an increase in the proportion of LPS-containing bacteria in the gut (40). *Zymomonas mobilis* integral fermentation broth, which was incubated at 30°C for 72 h to reach the count of 10^9^ CFU/mL but the metabolites is unclear, is effective in regulating intestinal transit, and the cell-free fermented broth is efficient in reducing cholesterol, LDL and very low density lipoprotein (VLDL) (69). Supplementation with probiotics has been shown to contribute to improving lipid metabolism through bile acid hydrolase (70, 71). And different strains of *L. plantarum* have different cholesterol-lowering capacities and different influencing factors (72). High-dose composite probiotics from camel milk, which include *Lactobacillus kefiranofaciens*, *L. plantarum*, *Lactobacillus helveticus*, *Lactococcus lactis* each 1.0 × 10^10^ CFU/mL and *Issatchenkia orientalis* 1.0 × 10^8^ CFU/mL, decreased fasting blood glucose and body weight, increased C-peptide, modulated lipid metabolism and improved liver and kidney protected in-jury in db/db mice, which may be related to various probiotics acting through protecting the function of islets and regulating intestinal flora disturbance (41). In other words, lipids and their metabolites interact with the gut microbiota to relieve inflammation.

## Probiotics Diversely Involved in Lipid Metabolism to Improve Gut Health

With the in-depth study of the gut microbiota, more and more attention has been paid to probiotics. There are many types of probiotics isolated from various environments, such as *Bifidobacterium*, *Lactobacillus*, and *Bacteroides*, which can be involved in lipid metabolism in direct and indirect ways (7, 8, 42, 73, 74). Therefore, we mainly summarized the probiotics involved in lipid metabolism through cholesterol metabolism, short-chain fatty acids (SCFAs), interaction with gut microbiota to reduce oxidative damage and improve intestinal barrier.

### Involved in Cholesterol Metabolism *via* Bile Acid Cycle

Body cholesterol comes from diet and biosynthesis. Adults can synthesize cholesterol in various tissues except brain tissue, among which the liver and intestinal mucosa are the main sites of synthesis. Cholesterol homeostasis is vital for proper cellular and systemic functions. Disturbed cholesterol balance underlies not only cardiovascular disease but also an increasing number of other diseases such as neurodegenerative diseases and cancers (42, 75–77). Cholesterol can be converted into sterols/oxysterols and bile acids. Among them, bile acids combined with glycine or taurine, and then flow into the intestine through the gallbladder to participate in the emulsification of dietary fat and promote absorption. Conjugated bile acids first generate cholic acid and chenodeoxycholic acid under the action of intestinal bacterial bile acid hydrolase, and then generate 7-deoxycholic acid and lithocholic acid under the action of bile acid 7α dehydroxylase (78–80). Most of the bile acids in the intestine are actively absorbed and passively diffused into the liver through the portal vein, and the rest are excreted in the feces. Several probiotic strains have been extensively studied and their benefits to human health have been widely documented. For example, it has been reported that probiotics, such as *Bifidobacterium longum*, *Lactobacillus acidophilus*, *L. casei, L. plantarum*, exhibit bile salt hydrolases (42, 72–74, 80, 81). For example, *L. casei* pWQH01, which overexpresses *bsh*1 from *L. plantarum* AR113, appear to improve steatosis *in vitro* in a BSH-dependent manner (81). *Lactobacillus gasseri* BNR17 can inhibit the secretion of adiponectin and leptin, and reduce adipose tissue, while *L. gasseri* SBT2055 reduced mesenteric tissue mass, serum leptin levels and adipocyte size in lean Zucker mice (82, 83).

### Produce Short Chain Fatty Acids to Participate in Lipid Metabolism

SCFAs are the products of otherwise indigestible fiber-rich diets metabolized by the anaerobic intestinal bacteria in the colon, mainly including formic acid, acetic acid, propionic acid, butyric acid, and valeric acid (19). Acetate is the most abundant and it is used by many gut commensals to produce propionate and butyrate in a growth-promoting cross-feeding process (84). A randomized double-blind show that probiotic *L. plantarum* IS 10506 supplementation significantly influenced acetate titer, but marginally significant for propionate and butyrate of women with functional constipation in the community of Jakarta (85).

SCFAs regulate lipid metabolism and play a role in immunomodulatory through a variety of ways. Firstly, SCFAs can activate the liver cyclic adenosine monophosphate (cAMP) signaling pathway to enhance the body’s oxidative metabolism, inhibit liver fat production, and thereby improve blood lipid levels. For example, Butyric acid regulates progesterone and estradiol secretion *via* cAMP signaling pathway in porcine granulosa cells (86). SCFAs can be recognized by G protein coupled receptors (GPRs), such as GPR41 and GPR43, which are expressed in colon, adipose, muscle, and liver tissue. Both GPR41 and GPR43 can reduce the concentration of intracellular cAMP, but GPR43 also increase the concentration of Ca^2+^, thereby inhibiting the synthesis of cholesterol in the liver to regulate lipid metabolism (87). SCFAs have been shown to ameliorate diseases in animal models of IBD and allergic asthma (88). Asparagus officinalis polysaccharides fermented with *L. plantarum* NCU116 can modulate the disordered homeostasis of bile acids, which is attributed to the up-regulation of GPR41 and GPR109A as well as intestinal SCFA production (89). Phenyl lactic acid derived from *L. plantarum* ZJ316 significantly increases the concentrations of propionic acid and butyric acid in the colon, regulates microbiota composition and alleviates *Salmonella enterica Typhimurium*-induced colitis (90).

Secondly, SCFAs have been shown to stimulate gastrointestinal chromaffin cells to secrete leptin, glucagon-like peptide-1 (GLP-1) and gastrointestinal peptide hormone tyrosine (PYY), thereby activating the afferent pathway of the vagus nerve to inhibits food intake (91–93). SCFAs can be used as a colonocyte energy source in the rat, and their derivatives produced by the digestion of intestinal flora can stimulate the secretion of PYY and GLP-1 *via* bind to GPR43, and inhibit the emptying of gastric contents (92). The secretion of gastric acid reduces appetite and suppresses eating, thereby alleviating the occurrence of hyperlipidemia. In addition, the combination of SCFAs and GPR43 promotes the secretion of GLP-1, up-regulates the expression of fat β-oxidation genes, promotes lipolysis, and avoids metabolic diseases such as hyperlipidemia caused by fat accumulation (93). Some SCFAs are associated with hepatic lipid metabolism and C-reactive protein concentrations, which may vary with gestational weight. Obesity in pregnancy reduces the amount of SCFAs in the stool, and a decrease in the level of butyrate reduces liver function (94, 95). Sodium propionate and sodium butyrate has been reported to counteract inflammation activation in the central and enteric nervous system (84, 96).

Moreover, SCFAs can also affect lipid metabolism by increasing the host’s sense of satiety and controlling appetite. SCFAs can target fat cells and increase the release of leptin, which has the effect of reducing hunger and inhibiting feeding activities (91, 94). Human milk insulin and leptin are independently associated with beneficial microbial metabolic pathways predicted to increase intestinal barrier function and reduce intestinal inflammation (97). Despite GPR43 being activated by SCFAs, it remains unclear if this receptor plays a role in leptin expression, as this receptor is expressed at low levels in adipocytes (43). While SCFAs stimulated leptin expression by activation of GPR41, which is highly expressed in adipose tissue (29). In animal studies, probiotics interventions to modulate high-fat diet outcomes predominantly caused a decrease in circulating leptin levels and increased SCFA, associated with suppressed weight gain (20). Fecal microbiota transplantation from Kazak individuals with normal glucose tolerance could improve glycolipid disorders by changing the bacterial composition responsible for producing SCFAs and activating the GPR43/GLP-1 pathway in db/db mice (98). In conclusion, probiotics mediate SCFAs production and regulate lipid metabolism in multiple ways.

### Regulate Gut Microbiota to and Strengthen Intestinal Barrier

Gut microbes are increasingly linked to human health. Under the influence of certain factors, the intestinal micro-ecosystem is destroyed, resulting in dysbiosis. Gut microbial dysbiosis contribute to obesity, *Clostridium difficile* infection, autoimmune disorders, IBD, and even bowel cancer (99, 100). Many studies have reported that the intake of exogenous probiotics and their products can improve the intestinal microbiota of animals and humans (42, 43). Probiotics can compete with pathogenic bacteria for space occupation and nutrients, and their metabolites can inhibit the growth of pathogenic bacteria, thus playing a certain protective role in the intestinal tract (23, 24, 90). Probiotics containing *B. animalis* subsp. *Infantis* BLI-02, *B. breve* Bv889, *B. bifidum* VDD088, *B. animalis* subsp. *lactis* CP-9, and *L. plantarum* PL-02 intervention changed the species, dispersion, and abundance of *gut macrobiota* and exhibit anti-oxidative activities that regulate oxidative stress and protect cells from oxidative damage in middle aged mice (20). However, the metabolites of opportunistic pathogens would lead to the oxidation of lipid metabolites and promote the development of intestinal inflammation. For example, *Aggregatibacter actinomycetemcomitans* could markedly promote the oxidation of LDL through oxidative stress involving NADPH oxidase- and myeloperoxidase-derived reactive oxygen species (101). Lipopolysaccharides (LPSs) are cell wall components of Gram-negative bacteria, also called endotoxins. Normally, they are confined to the intestinal lumen and cannot cross the intestinal barrier. However, when the intestinal barrier function is weakened, LPSs can enter the bloodstream and cause systemic inflammation. Elevated concentrations of LPS in circulation may increase risk of atherosclerosis (102). While *L. casei* has been shown to destroy the biofilm of *Proteus mirabilis* P14 to avoid damage to the gut barrier (103).

In the food industry, there are some probiotics that can modulate immune response are widely used, such as *Lactobacillus johnsonii*, *Lactobacillus rhamnosus*, *L. casei*, *Saccharomyces cerevisiae*, *Bifidobacterium lactis*, and *Bifidobacterium animalis*. It was reported that *Pediococcus pentosaceus* CECT 8330 administration protects the DSS-induced colitis and modulates the gut microbial composition and function, immunological profiles, and the gut barrier function (43). Numerous clinical evidence and scientific literature have shown that the use of *L. rhamnosus* LGG^®^ alone is associated with improved immunity, while the combined use of *Bifidobacterium lactis* BB-12™, *Bifidobacterium infants* DSM 33361 and *Streptococcus thermophilus* TH-4™, significantly reduced necrotizing enterocolitis in preterm infants (29). In addition, probiotics also exhibited strain-specific protective effects in T84 cells challenged with *C. difficile*-infected fecal water, whereby *Saccharomyces boulardii* CNCM I-1079 and *L. rhamnosus* R0011 were most effective in reducing pro-inflammatory cytokine production, while *L. rhamnosus* GG R0343 along with the two other multi-strain combinations were the least effective (21). Probiotics can reduce inflammation by exerting antioxidant properties. For example, the protective effect of Bifidobacterium infantis BB on allergic mice was correlated with its antioxidative enzyme SOD (104). The study demonstrates that *Lactobacillus sakei* pro-Bio65 empowers the antioxidant endogenous efficiency of HCE cells, by the upregulation of the glutathione (GSH) content and the enzymatic antioxidant pattern, and concurrently reduces TNF-alpha protein expression (105). Generally speaking, probiotics can regulate gut microbiota, reduce lipid peroxide damage, and strengthen the immune barrier.

## Conclusion

IBD and bowel cancer are serious worldwide health problems that are influenced by lipids metabolites and gut microbes. Lipids and their metabolites interact with gut microbiota, which is influenced by supplemental probiotics. The metabolites of triglycerides, cholesterol lipids, and phospholipids can be converted into each other. In this review, we focus on the interconnectedness of triglycerides and cholesterol and their metabolites with gut microbes and IBD. Among them, the derivates of ω-6 PUFAs AA LTs and PGs can promote the production of pro-inflammatory factors such as TNF-α, IL-1β, IL-6, and IFN-γ. While ω-3 PUFAs DHA, EPA and their derivatives RvE1, RvDs, and protectin D would increase the expression of anti-inflammatory factors IL-10 and IL-4. Cholesterol is often present in the blood in the form of VLDL, LDL, and HDL. LDL is easily oxidized to oxLDL and promotes inflammation, but HDL can play an anti-inflammatory function. When UC recurs over a long period of time, it increases the risk of developing bowel cancer. Probiotics such as *Bifidobacterium*, *Lacticaseibacillus*, and *Lactiplantibacillus*, participate in lipid metabolism by regulating intestinal flora, producing SCFAs and BSH. And they produce SOD and GSH, which effectively alleviate oxidative stress damage and inhibit the development of inflammation. There is increasing evidence that probiotics have the potential to improve overall health. However, this research area needs to be thoroughly examined in more clinical studies to further explore the health-promoting effects and mechanisms of probiotics in IBD and bowel cancer. Therefore, it has broad application prospects for the prevention and control of the occurrence and development of intestinal cancer by regulating the intestinal microecology through personalized probiotics.

## Author Contributions

TW: conceptualization and writing. GW: resources and review. ZX, HZ, and YW: resources. YX and XS: project administration. LA: funding acquisition. All authors have read and agreed to the published version of the manuscript.

## Conflict of Interest

The authors declare that the research was conducted in the absence of any commercial or financial relationships that could be construed as a potential conflict of interest.

## Publisher’s Note

All claims expressed in this article are solely those of the authors and do not necessarily represent those of their affiliated organizations, or those of the publisher, the editors and the reviewers. Any product that may be evaluated in this article, or claim that may be made by its manufacturer, is not guaranteed or endorsed by the publisher.
